# The crucible of COVID-19: what the pandemic is teaching us about health research systems

**DOI:** 10.1186/s12961-020-00573-1

**Published:** 2020-06-01

**Authors:** Tari Turner, Fadi El-Jardali

**Affiliations:** 1 Monash University, Melbourne, Melbourne, Australia; 2 American University of Beirut, Beirut, Lebanon

**Keywords:** COVID-19, coronavirus, research, systems

## Abstract

The global health crisis created by COVID-19 is providing valuable insights into the strengths of our health research system and, perhaps even more clearly, displaying its weaknesses. Much of what is being shown so plainly in the current context is not truly new. We are being reminded that health research systems are slow and noisy as well as that there is a desire for research to inform decision-making, that researchers are great collaborators, and that the walls we are so quick to erect between health research and health practice are unhelpful facades. It is our hope that the clarity with which these issues are being demonstrated by COVID-19 might provide the impetus to address these challenges and seize these opportunities to improve our health research system, for the benefit for communities facing COVID-19 now, and for the benefit of us all in facing the further health challenges that are sure to come.

## Main text

*“We are only what we always were”* — Arthur Miller, The Crucible.

If it is true that, under pressure, we all revert to type, then COVID-19 is revealing quite a few flattering things about the true nature of the health research community. It turns out that, when the going gets tough, researchers get productive, collaborative and impact focused. It is as if the nature of this current and unprecedented crisis is reminding us of all the reasons for which we embarked on research careers; that we are now seeing afresh that research is vital, is valuable and can be truly lifesaving, and we are kicking research production up a gear.

Sadly, we are also seeing that the systems we have in place to translate the results of research into improvements in health practice and policy are not fit for purpose in a pandemic. If we are frank, there is little in our experience of knowledge translation with COVID-19 that is truly new; mostly what we are seeing are the known strengths and weaknesses of our system held up to a very, very clear light. So, what is COVID-19 revealing?

### Research publication is slow

The current system for publication of peer-reviewed research is slow. This is not news. However, in the light of COVID-19, it is very clear that the delays caused by standard workflows, which are both appropriately rigorous and inappropriately bureaucratic, are slowing the translation of new research evidence into practice, and that lives are being lost in those lost days, weeks and months.

A now familiar example is a key paper on the effectiveness of face masks [1], for which *Nature Medicine* achieved what would have, in other times, been considered a sprightly turnaround, but which resulted in a 1-month delay between receipt of the paper and its publication. The window between acceptance and publication was a full 2 weeks. Realising the importance of publication speed, some journals and publishers are implementing rapid publication models and many research teams are implementing workarounds to make research available more quickly, making prepublication versions of reports available, creating online repositories of rapid reviews and sharing emerging data.

These are useful and well-intentioned steps; it will be important that, in a post-pandemic world, we seize on these initiatives, refine them and, where possible, scale them up. However, we need to acknowledge that they are creating flow-on problems, research that has not been peer-reviewed is used as the basis of decision-making, finding and curating all of those duplicating and overlapping reviews is very challenging, and how data has been validated and analysed is often unclear.

Interestingly, research platforms that were designed to overcome some of these challenges in a pre-pandemic world, like *F1000*, have not been swamped by publications. If this was their moment, the research community does not seem to have realised it. On the other hand, the growing interest in ‘living’ continually updated evidence reviews does seem to have taken off in an environment where so little is certain and new evidence is available every day.

### Research publication is noisy

Over the first few months of 2020, there has been an initially small, but dizzyingly rapid production of primary research and reviews of research related to COVID-19. These research studies have been broadly disseminated through the usual research publication systems as well as through social media, clinical networks and other channels. Challengingly, this useful research has been accompanied by a blizzard of commentary, opinion and editorialising; often from the same publication platforms as the research, but also from everyone else, up to and including celebrity chefs.

Clinicians, managers and policy-makers (and journalists) who were valiantly trying to stay across all the research as it emerged were quickly overwhelmed by the confetti of competing, unsubstantiated, conflicting voices and views, all purporting to be providing ‘evidence’. We need better strategies to remove the noise from our research publication and dissemination systems, and we need them now. Simple flags for identifying research that has been peer reviewed or accessible, curated collections of rapid systematic reviews would be useful steps in the right direction.

### There is a desire for research

Having been increasingly disheartened by the cynical attitudes towards ‘experts’ and ‘evidence’ so sadly ubiquitous in the pre-COVID-19 world, it has been hugely encouraging to see the widespread demand for research evidence to guide health decision-making at all levels, from individual patients to global multi-laterals.

This presents a huge opportunity for the research community to demonstrate their ability and willingness to engage, and it has been encouraging to see our colleagues step up and into the conversation. There is no doubting the real-world impact of research on decision-making in the shadow of COVID-19, even if it will be months before we see the publication metrics.

The real challenge will be converting these acute ‘evidence hunger pangs’ into an ongoing appetite in our governments, health systems and others to fund and use research evidence to guide decision-making beyond the pandemic. To achieve this, we need to ensure that we capture data and stories of the impact of research on decision-making during the current crisis, so we can build on this momentum following the pandemic.

### Researchers are collaborators

Never before have so many researchers asked for access to pre-publication data from their colleagues on the other side of the globe and received a rapid and resounding ‘yes’. COVID-19 has provided an undeniable imperative to put aside concerns of being pipped to the publishing post or to the funder’s wallet, to collaborate in the service of the global, common and immediate good.

As elsewhere, our challenge as a research community is to translate this willingness to share beyond the bubble produced by this pandemic and beyond the espoused rhetoric; to build and implement models for research funding, conduct and publication that reward true collaboration and acknowledge the reality that the partnerships required for successful collaborative research are complex and time consuming as well as, ultimately, incredibly rewarding.

### It is all one evidence ecosystem

The line between research and practice has always been marked in fuzzy pencil and COVID has taken a big smudgy eraser to that already unclear demarcation. A novel virus, an unprecedented health emergency, a non-existent evidence base. In this evidence-free zone, when does a clinical hunch, a series of cases, the experience of a hospital for a month, become the best available evidence? When do we wait for the results of the randomised trial possibly months away and when do we make recommendations based on early indications from an ICU in China? COVID-19 has already demonstrated that, sometimes, early indications are very misleading and at others they are crucial. Hydroxychloroquine, which was touted early in the outbreak as a revolutionary treatment, is looking less and less promising as rigorous trials emerge [2] and clinical experience has been key in identifying that the symptoms of COVID-19 may deteriorate rapidly on days 6–10 of illness, life-saving information for ensuring patients are appropriately monitored even if apparently well [3]. We need to stop pretending that there is clinical and public health expertise that is entirely distinct from research knowledge and we need to build our methods for integrating the two in pragmatic and transparent ways.

We also need to accept that a range of contributors are all crucial in producing the knowledge we need to effectively deal with this most challenging foe. Vital knowledge about COVID-19 has been produced along the entire research spectrum from the very fundamental, ‘basic’ research to the most hands-on clinician researcher; the engine rooms of translation have been the multidisciplinary networks of clinical specialists, policy-makers and researchers in formal discussion lists, on knowledge translation platforms and in professional societies. Community organisations, citizens and the media have all played important roles in identifying issues and disseminating information in (socially distanced) corridor chats, tweets and WhatsApp messages. As is always true, and is even more so now, there is no us and them, we are all us.

### Conclusion: the possibilities

It has already become a cliché that COVID-19 presents us with a pivotal moment; there is no question that many decisions made now will have a huge impact on history, for better or for worse. For health research systems, this is also true. We are in a moment where, if we do not let it consume us, we can use the light cast by this inferno to see ourselves, our strengths and our weaknesses, clearly. This crucible of COVID-19 highlights the fundamental need to institutionalize the use of research evidence in policy decisions at a time of crisis, and beyond. If we act now, we might even emerge stronger and with greater purpose than when we entered in this horrific conflagration.

## Data Availability

Not applicable.
